# Towards an Adoption Framework for Patient Access to Electronic Health Records: Systematic Literature Mapping Study

**DOI:** 10.2196/15150

**Published:** 2020-03-30

**Authors:** Hugo J T van Mens, Ruben D Duijm, Remko Nienhuis, Nicolette F de Keizer, Ronald Cornet

**Affiliations:** 1 Department of Medical Informatics Amsterdam Public Health Amsterdam University Medical Centers, University of Amsterdam Amsterdam Netherlands; 2 Department of Research & Development ChipSoft Amsterdam Netherlands

**Keywords:** electronic health records, evaluation studies as topic, personal health records, patient access to records, patient portals

## Abstract

**Background:**

Patient access to electronic health records (EHRs) is associated with increased patient engagement and health care quality outcomes. However, the adoption of patient portals and personal health records (PHRs) that facilitate this access is impeded by barriers. The Clinical Adoption Framework (CAF) has been developed to analyze EHR adoption, but this framework does not consider the patient as an end-user.

**Objective:**

We aim to extend the scope of the CAF to patient access to EHRs, develop guidance documentation for the application of the CAF, and assess the interrater reliability.

**Methods:**

We systematically reviewed existing systematic reviews on patients' access to EHRs and PHRs. Results of each review were mapped to one of the 43 CAF categories. Categories were iteratively adapted when needed. We measured the interrater reliability with Cohen’s unweighted kappa and statistics regarding the agreement among reviewers on mapping quotes of the reviews to different CAF categories.

**Results:**

We further defined the framework’s inclusion and exclusion criteria for 33 of the 43 CAF categories and achieved a moderate agreement among the raters, which varied between categories.

**Conclusions:**

In the reviews, categories about people, organization, system quality, system use, and the net benefits of system use were addressed more often than those about international and regional information and communication technology infrastructures, standards, politics, incentive programs, and social trends. Categories that were addressed less might have been underdefined in this study. The guidance documentation we developed can be applied to systematic literature reviews and implementation studies, patient and informal caregiver access to EHRs, and the adoption of PHRs.

## Introduction

Patient access to electronic health records (EHRs) is becoming increasingly common and is even a legal right in many countries. EHRs have been associated with increased patient engagement and improved health care quality outcomes [1-8]. However, there are also barriers to patients’ access to EHRs. For example, some patients have difficulties logging in to patient portals and personal health records (PHRs), which facilitate access, due to complicated security procedures [1-8]. A framework is needed to assess the determinants and outcomes of PHR and EHR adoption that facilitates this access. This framework should consider patients and informal caregivers as users rather than health care providers alone. This framework would enable the comparison and aggregation of evidence, and provide an overview of any important factors involved, which can then be used as a guide in implementations and health care policies as well as to address the gaps in knowledge.

**Figure 1:**
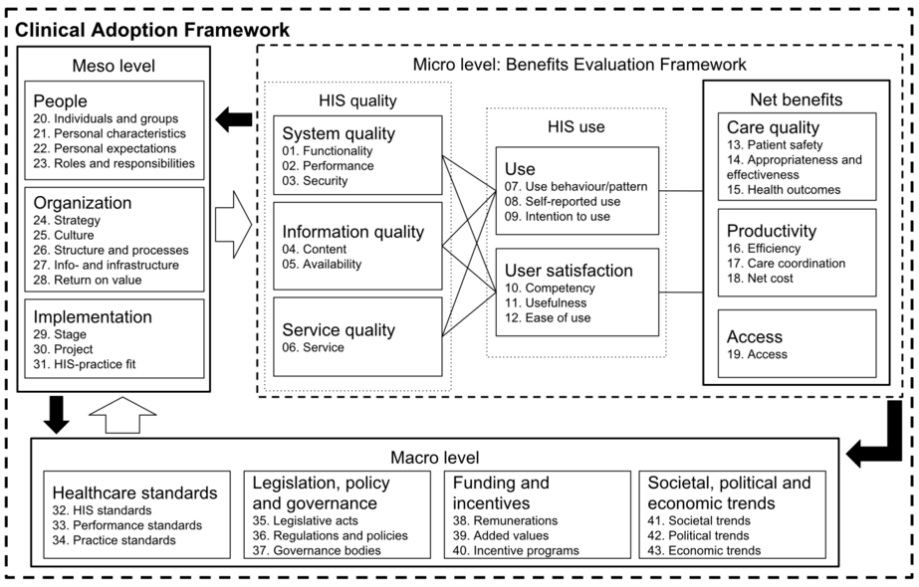
Clinical Adoption Framework with levels, dimensions, and categories. HIS: health information system. Originally published in [10, 18].

“The Clinical Adoption Framework (CAF) is a general evaluation framework to assess the success of health information system (HIS) adoption in healthcare organisations” [9,10]. PHRs and EHRs are types of HISs, and thus this framework is also applicable to them. “As shown in Figure 1, it addresses the micro level, which encompasses the dimensions of quality, use and net benefits of the HIS; the meso level, consisting of the dimensions people, organisation and implementation; and the macro level, incorporating the dimensions healthcare standards, legislation, policy and governance, funding and incentives, and societal, political and economic trends” [10]. Within each dimension, several categories were distinguished, for example “01. Functionality”, “02. Performance”, and “03. Security” are categories of the dimension “HIS quality” at the micro level. “It is hence an integrated framework that covers a wide range of aspects involved in HIS adoption” [10]. The CAF was developed and validated through consultation with health information technology professionals, comparisons with other survey instruments, and a meta-review of 50 systematic reviews on HIS implementation [11]. Categories, dimensions, and levels of the CAF were originally described by Lau, Price, and Kashevjee [9]. Throughout the categories, dimensions, and levels there are feedback loops, which are indicated by the arrows in Figure 1, that resembles the interplay between the factors and nondeterministic characteristics of HIS adoption and the outcomes of HIS use [9,11]. The CAF was applied in over 30 studies [12-18].

The CAF is a complex framework consisting of 43 categories that belong to 15 dimensions (illustrated as small boxes in Figure 1), which are further separated into the 3 previously mentioned micro, meso, and macro levels [9]. The CAF was considered difficult to apply as there was no guidance documentation with explicit descriptions and rules regarding its use [11]. Consequently, studies [15-18] that have applied the CAF differed in their interpretations and applications. Furthermore, HIS adoption increasingly involves sharing medical data with patients and informal caregivers. Therefore, patients and caregivers should also be considered when understanding successful HIS implementation, because they might value different factors than health care providers. This patient and caregiver perspective was not explicitly taken into account during the development of the CAF.

The primary objective of this study was to extend the CAF to make it useful for evaluating patients' access to EHRs and the adoption of PHRs. The second objective was to improve the consistent application of the CAF in literature and implementation studies. For this purpose, we aimed to assess the interrater reliability of applying the framework, discuss which areas of the CAF could be improved, and develop guidance documentation.

## Methods

We systematically reviewed existing systematic review papers on determinants and outcomes of patients’ access to their personal health data. Results from each review paper were mapped to categories in the CAF, which was adapted when needed to reach consensus. The protocol for this review study was developed using the first 6 review papers [19-24], which were the most recent publications at the beginning of this review study. We used 13 subsequent review papers [25-37] in this study to refine the CAF and to assess the interrater reliability. The review protocol was registered at PROSPERO under CRD42018084542 [38]. We then reported the results of adapting the CAF, including its reliability, to make it suitable for an evaluation of the adoption of PHRs and patients’ access to EHRs. The results of the review study on the determinants and outcomes of patients’ access to medical records were reported separately [10].

**Figure 2:**
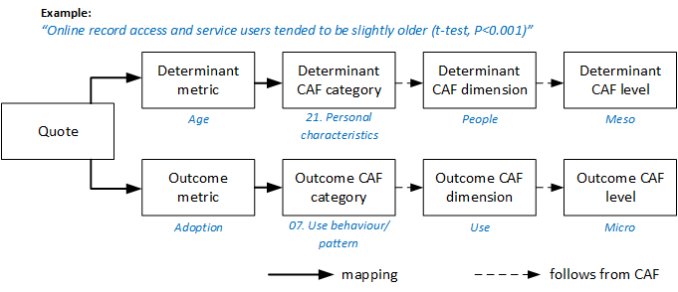
Example of how a quote is mapped to a determinant metric and outcome metric. CAF: Clinical Adoption Framework.

To improve the CAF and its definitions, one reviewer (HM) extracted quotes from the literature that described determinants and outcomes for the adoption of EHRs and PHRs, and another reviewer (RD) verified these extracted quotes. The two reviewers independently mapped the extracted quotes. The interrater reliability for the agreement on the mapping was calculated with Cohen’s unweighted kappa [39,40]. Each quote was mapped to two CAF categories: one for the determinant of the quote and the other for the outcome. Within each category, the quotes were classified into metrics by thematic analysis, as illustrated in Figure 2. The metrics and categorizations were iteratively revised to ensure consistent and meaningful categories for summarizing results, which was similar to the process described in Bassi, Lau, and Lesperance [15]. The mapping to two categories is visualized in Figure 2. For example, in the quote “Online record access and service users tended to be slightly older (*t*-test, *P*<0.001)” [31], the determinant metric could be “Age” and the outcome metric “Adoption”. Age would be classified as “21. Personal characteristics”, under the dimension “People” at the meso level, while adoption would be classified as “07. Use behaviour/pattern”, under the dimension “Use” at the micro level. For the sake of the review, we added the category “44. Other” to denote when a quote could not be classified using the CAF.

The results of this mapping and the differences in quote interpretation and CAF categorization were discussed among the two reviewers to achieve consensus. When necessary to achieve consensus, the definitions of the CAF were adapted and extended with inclusion and exclusion criteria to make them clearer. We presented the number of definitions for categories that were introduced, extended, or unchanged in each level. For agreements and disagreements between reviewers on mapping quotes to categories, we calculated the number of times each unique combination was agreed or disagreed upon (ie, number of times there was agreement on one certain category or disagreement between two specific categories). We counted the number of quotes that were classified into each category by a reviewer as well as how many quotes could not be mapped to the CAF. The level of agreement between reviewers on mapping quotes to each category indicated how ambiguous or well-defined the category was. This process resulted in defined categories of the CAF with inclusion and exclusion criteria and a list of metrics that we distinguished. Statistical analysis was carried out in R version 3.5.0 (R Foundation for Statistical Computing, Vienna, Austria) with RStudio 1.1.453 (RStudio Inc, Boston, MA). The R script can be found in Multimedia Appendix 1.

## Results

In this section, we first list the definitions that were unchanged, extended, or introduced. Second, we discuss the interrater reliability and the spread of mapping quotes to CAF categories.

### Adaptation of CAF Categories and Found Metrics

Definitions were introduced to the CAF for the 19 micro level categories, because they were missing in the original publication of the CAF. For example, the category “01. Functionality” of the dimension “System quality” was defined with the inclusion criteria “Actual or missing features/functionalities of the HIS and their quality” and the exclusion criteria “If adoption or use of the HIS in general, without a particular functionality, then choose 07. Use behaviour/pattern”. Thus, the exclusion criteria were made explicit for when a quote must be classified in another category. For the 24 meso- and macro level categories, the definitions from Lau, Price, and Kashevjee [9] were used, either unchanged (9 categories) or extended (15 categories), to cover cases of patient and informal caregiver use and disambiguate the categories with refinements and exclusion criteria. For example, the definition for the category “21. Personal characteristics” of the dimension “People” was extended with “socio-economic status, ethnicity, computer skills, (health) literacy, health status” and “Behaviour”. These are factors that were found to be important for the adoption of a HIS by patients and caregivers, and were not included in the original CAF category definition. Table 1 shows the number of categories for each level and how many were introduced, extended, or unchanged. Table 2 shows the categories that were changed and provides an example for each level. The resulting definitions for disambiguation in each category are listed in Table A in Multimedia Appendix 2. The metrics of each category can be found in Table B in Multimedia Appendix 2.

**Table 1:** The number of categories with introduced, extended, and unchanged definitions per level.

Level	Introduced	Extended	Unchanged	Total
Micro	19	0	0	19
Meso	0	9	3	12
Macro	0	6	6	12

**Table 2:** Categories where inclusion and exclusion criteria were added.

Level	Categories changed	Example [additions in brackets]
Micro	All categories from “01. Functionality” to “19. Access”	Inclusion criteria introduced for “01. Functionality”: [Actual or missing features/functionalities of the HIS^a^ and their quality.] Exclusion criteria introduced for “01. Functionality”: [For adoption or use of the HIS in general, not a particular functionality, use category “07. Use behaviour/pattern”]
Meso	“20. Individuals and groups” “21. Personal characteristics” “22. Personal expectations” “23. Roles and responsibilities” “25. Culture” “27. Info- and infrastructure” “28. Return on value” “30. Project” “31. HIS-practice fit”	Inclusion criteria extended for “21. Personal characteristics”: “Degree to which an individual’s characteristics, such as age, gender, education, [socio-economic status, ethnicity, computer skills, (health) literacy, health status,] experience and expertise can affect the adoption of an HIS” [9]. [Behaviour].
Macro	“35. Legislative acts” “36. Regulations and policies” “39. Added values” “41. Societal trends” “42. Political trends” “43. Economic trends”	Definition extended with exclusion criteria for “35. Legislative acts”: [For privacy concerns use category “22. Personal expectation.”]

^a^HIS: health information system.

### Interrater Reliability and Spread

From the 13 reviews [25-37], we extracted 624 quotes. Each of the 624 quotes were mapped twice (ie, to a determinant and an outcome category) resulting in 1248 mappings. We achieved a percentage agreement of 67.0% (418) and a kappa of 0.58 for the determinant category, and a percentage agreement of 62.5% (390) and a kappa of 0.55 for the outcome category. As shown in Table C and Table D in Multimedia Appendix 2, the three categories that were least ambiguous, based on their high agreement scores, were “16. Efficiency”, “21. Personal characteristics”, and “13. Patient safety”. In contrast, categories “09. Intention to use”, “04. Content”, and “30. Project” showed low agreement scores. Some disagreements between two categories occurred more often than others. For example, a feature relating to secure messaging or access to medical records was interpreted by one reviewer as “’01. Functionality” and the other reviewer as “’07. Use behaviour/pattern” 94 times. This happened for instance with the quote “Patients experienced easier communication and interactive discussion with their physician after reading the medical file” [37]. There was one quote that did not fit into any one of the categories: “Two articles proposed achieving data exchange by setting up (Regional) Health Information Exchanges that can standardize data and facilitate exchange among different organizations.” [24]. This result referred to infrastructure that exists outside of an organization to facilitate data exchange between organizations and would, therefore, fall into the macro level.

## Discussion

### Principal Findings

The definitions of the CAF categories were extended to be applicable to patient access to EHRs and the adoption of PHRs. This was achieved by adding factors that were found in the reviewed literature on patient access to EHRs, but were not present in the CAF yet, as was illustrated in the example of “21. Personal characteristics” in the results section. In addition, we developed guidance documentation in the form of inclusion criteria, exclusion criteria, and a list of metrics found. The interrater reliability of the reviewers applying the adapted CAF was moderate. However, we found the CAF to be a highly suitable and comprehensive framework to address patients’ access to EHRs, as we could achieve consensus on the mappings through discussion, and almost all results could be categorized in the CAF. The original content for the definitions of the CAF were unchanged and only extended with additional inclusion and exclusion criteria for disambiguation and for the application to patients’ access to EHRs. The number of agreements and disagreements and percentage of agreements varied among the CAF categories, just like the number of quotes that were mapped to each category. Some categories were not found at all in the reviews, especially those on the macro level.

### Strengths and Weaknesses of the Study

We showed how the CAF can be applied to studies evaluating patient access to EHRs and PHRs. Despite many publications on the application of the CAF, we are the first, to our knowledge, to provide measures on the interrater reliability. However, the unweighted Cohen’s kappa does not consider that categories actually reflect an order and results within each review are all correlated and come from the same study. Nonetheless, the moderate agreement indicates that the extended CAF is applicable in a consistent way. Because this study was a systematic review of systematic reviews, we have not investigated how to apply these results in primary implementation studies. The categories that were mapped to a lesser extent might have been underdefined, especially those at the macro level. It is possible that these categories may not have been reported in the literature, but also the literature may not have addressed the topics from those categories, or those categories could have been reported in other types of literature such as in policy, law, or gray literature, rather than scientific medical literature. Those categories with relatively high disagreement should also be further evaluated and redefined. Furthermore, the CAF could be used in studies to present their results in a more structured and standardized way. This will improve the ability to compare the results of different studies.

### Results in Relation to Other Studies

The variability in the application of the CAF categories found in previous studies [15-18] can be explained by ambiguities that were addressed by the inclusion and exclusion criteria of this study. In addition, we found that mapping to a determinant and an outcome CAF category, instead of only one, decreased some of the ambiguity. Only one result, concerning regional information exchange, could not be mapped in the original CAF. This shows that overall the CAF is sufficiently comprehensive. However, we believe that the infrastructure that is available in the environment of an organization forms a missing category in the framework. This category could be introduced in the framework at the macro level to incorporate regional information and communication technologies (ICT) infrastructure, which might be more advanced in some regions than in others.

### Implications of the Study

This adapted framework can be used in other reviews and in implementation studies of HISs, especially when the HIS has patients and informal caregivers as users. The definitions and metrics provided will still be of value to implementation studies by pointing out several aspects and metrics that have to be considered when carrying out HIS implementations. Furthermore, the results of this study fulfill part of the need for more guidance documentation when applying the CAF [11]. Our definitions with inclusion and exclusion criteria as well as the metrics found may contribute to a more consistent application of the framework. We recommend addressing specific relationships between determinants and outcomes using this framework, as we did by mapping quotes from the literature to two CAF categories.

### Conclusions

The scope of the CAF was extended to the adoption of PHRs, in addition to EHRs, by health care providers, patients, and informal caregivers. Further definitions and inclusion and exclusion criteria disambiguate and guide the application of each category. We found moderate interrater reliability in applying the framework and variance among the categories in the framework. Future research should address the application of the CAF in primary implementation studies and studies focusing on macro level topics such as international and regional ICT infrastructures, standards, politics, incentive programs, and social trends.

## Appendix

Multimedia Appendix 1 R script with syntax for analysis.

Multimedia Appendix 2 Supplementary tables.

## Abbreviations

CAF: Clinical Adoption Framework
EHR: electronic health record
HIS: health information system
ICT: information and communication technologies
PHR: personal health record.
