# Supplementary material for: Towards an Adoption Framework for Patient Access to Electronic Health Records: Systematic Literature Mapping Study
Source: JMIR Med Inform. 2020 Mar 30;8(3):e15150. doi: 10.2196/15150 (PMC7154932; doi:10.2196/15150)
Supplement: Multimedia Appendix 2 [file medinform_v8i3e15150_app2.docx]

Multimedia Appendix 2. Supplementary tables

**Date:** 4-11-2019

**Authors:** Hugo J.T. van Mens^a,b,^^[[1]](#footnote-1)^, Ruben D. Duijm^a,b^, Remko Nienhuis^b^, Nicolette F. de Keizer^a^, Ronald Cornet^a^

^a^ Amsterdam UMC, University of Amsterdam, Department of Medical Informatics, Amsterdam Public Health, Meibergdreef 9, Amsterdam, Netherlands

^b^ Research and Development, ChipSoft B.V., Amsterdam, The Netherlands

Appendix of “Towards an Adoption Framework for Patient Access to Electronic Health Records”.

Contents

[Table 1: Adapted CAF 2](#_Toc23773743)

[Table 2: Metrics per CAF category 14](#_Toc23773744)

[Table 3: Agreement per CAF category 22](#_Toc23773745)

[Table 4: Agreement per CAF category sorted by percentage agreement 24](#_Toc23773746)

## Table A: Adapted CAF

The purpose of this table is to classify a quote as determinant or outcome factor within the CAF. To this end, we defined in- and exclusion criteria. Inclusion criteria clarify what factors are included in the particular category and the exclusion criteria contain rules on when to classify a factor into another category. This way categories were disambiguated. The CAF is only used to classify determinants and outcomes of HIS adoption. Therefore, we used the ‘00. Exclude’ category to exclude quotes that were mistakenly taken under review, and were found not to be actual determinant or outcome factors after further evaluation and discussion. Further details on the procedure to apply the adapted CAF to literature studies can be found in the review protocol that was registered at PROSPERO under CRD42018084542.

Table A The Adapted Clinical Adoption Framework. It consists of levels, dimensions and categories. An “exclusion category” and an “other category” have been added for the mapping. For each category, inclusion and exclusion criteria have been provided. In the category column, the type of change of the in- and exclusion criteria (either introduced, extended or unchanged) is mentioned between brackets. Adapted from Lau (2011)^[[2]](#footnote-2)^

| Id | Level (change) | Dimension | Category (type of change of criteria) | Inclusion criteria | Exclusion criteria |
| --- | --- | --- | --- | --- | --- |
| 00 | Exclude | Exclude | Exclude (introduced) | Not actually a success factor (e.g. determinant, outcome, cost, benefit, barrier, facilitator, problem, solution, advantage, disadvantage, cause, effect, association) of using the HIS. Not actually a result of the review. Or it is unclear what is meant. |  |
| 01 | Micro | System quality | Functionality (introduced) | Actual or missing features/functionalities of the HIS and their quality. | Adoption or use of the HIS in general, not a particular functionality --> 07. Use behaviour/pattern |
| 02 | Micro | System quality | Performance (introduced) | Reliability, downtime, response time, technical issues and performance of the HIS. | Provider or support staff response time --> 06. Service. Internet speed -> 27. Info- and infrastructure. |
| 03 | Micro | System quality | Security (introduced) | Security of the HIS, including authentication and authorization performed by the system. | Login (authenticating yourself) -> 01. functionality. Concerns about security or privacy in general by an individual who has an effect on HIS adoption, not related to the usage of the particular HIS or their records in the particular HIS --> 22. Personal expectations. Security measures on organisational level --> 27. Info and infrastructure. Concerns about privacy in general in society; not related to the particular HIS or people who directly affect HIS adoption --> 41. Societal trends. |
| 04 | Micro | Information quality | Content (introduced) | Content, completeness, consistency, accuracy, intelligibility of the information in the HIS. | Organisational decisions, discussion or solutions on these issues --> 27. Info and infrastructure. |
| 05 | Micro | Information quality | Availability (introduced) | Timeliness, reliability, accessibility of records, and accessibility of the information in the HIS. | Organisational decisions, discussion or solutions on these issues --> 27. Info and infrastructure. |
| 06 | Micro | Service quality | Service (introduced) | Technical support for the use of the HIS and clinical, medical support to understand the information in the HIS. Response time of the service (by the healthcare provider or support staff), including response time to secure messaging. | System response time --> 02. Performance. Clinical content of messaging (e.g. clinical advice) --> 14. Appropriateness and effectiveness. |
| 07 | Micro | Use | Use behaviour/pattern (introduced) | Actual usage patterns and behaviour of the HIS measured objectively (not reported by the user itself, but e.g. by log files). Adoption of the HIS. | Self-reported use or subjective measures of usage --> 08. Self-reported use. A factor that influenced the usage --> 01-06. HIS quality or 20-43. Determinants. Particular functionality of the HIS --> 01. Functionality. |
| 08 | Micro | Use | Self-reported use (introduced) | Self-reported usage patterns and self-reported usage behaviour by the user of the HIS. | Actual usage patterns and behaviour of the HIS measured in another way, or unclear whether it was measured subjectively --> 07. Use behaviour/pattern. A factor that influenced the usage --> 01-06. HIS quality or 20-43. Determinants. |
| 09 | Micro | Use | Intention to use (introduced) | The actual intention to use or not to use the HIS or the HIS feature. | Believes and expectations about using HISs in by people who can affect the adoption of an HIS --> 22. Personal expectations. |
| 10 | Micro | User satisfaction | Competency (introduced) | Learnability, learning curve, competency and ability to understand and use the system. | Knowledge, experience or skills in general, not related to the particular HIS (implementation) used, e.g. medical knowledge or computer skills --> 21. Personal characteristics. Training provided to use the HIS --> 30. Project. User satisfaction --> 11. Usefulness. Ease of use and usability, Difficulty for the user to use the HIS --> 12. Ease of use. Awareness about HISs in general --> 22. Personal expectations. |
| 11 | Micro | User satisfaction | Usefulness (introduced) | User satisfaction about and usefulness of the HIS. | Expectations or user satisfaction about using HISs in general --> 22. Personal expectations. Ease of use --> 12. Ease of use. Degree of fit with daily life of patient or work processes --> 31. HIS practice fit. |
| 12 | Micro | User satisfaction | Ease of use (introduced) | Ease of use and usability of the HIS. | Learnability --> 10. Competency. User satisfaction --> 11. Usefulness. |
| 13 | Micro | Care Quality | Patient safety (introduced) | Patient safety, adverse events, risks or medical errors as an outcome of the usage of the HIS. |  |
| 14 | Micro | Care Quality | Appropriateness and effectiveness (introduced) | Effectiveness, guideline compliance or treatment adherence, appointment attendance, healthcare utilization as an outcome of the usage of the HIS. Knowledge or awareness about the condition or treatment, preparedness for doctor visits, participation in or engagement with healthcare as an outcome of the usage of the HIS. | Patient-provider engagement, participation or communication, self-management or healthcare accessibility --> 19. Access. |
| 15 | Micro | Care Quality | Health outcomes (introduced) | Health outcomes, medical outcomes, physiological outcomes, psychological outcomes and quality of life as an outcome of the usage of the HIS. | Health knowledge or awareness, patient engagement as an outcome of the usage of the HIS --> 14. Appropriateness and effectiveness. |
| 16 | Micro | Productivity | Efficiency (introduced) | Efficiency, resource utilization, time needed, workload as an outcome of the usage of the HIS. | As an incentive for the organisation that adopts the HIS --> 28. Return on value. As a general expectation on macro level --> 39. Added values. Appointment attendance --> 14. Appropriateness and effectiveness |
| 17 | Micro | Productivity | Care coordination (introduced) | Coordination of care, continuity of care, communication between providers, workflow as an outcome of the usage of the HIS. | Patient-provider communication and access to health care --> 19. Access. |
| 18 | Micro | Productivity | Net cost (introduced) | Financial costs and benefits as an outcome of the usage of the HIS. | Cost/benefit as an incentive/determinant that influences the HIS adoption on organizational level --> 28. Return on value. Remunerations available that influence the meso level (people, organisation, project) dimensions that affect clinical adoption --> 38. Remunerations. Incentive programs that influence the meso level (people, organisation, project) dimensions that affect clinical adoption --> 40. Incentive programs. |
| 19 | Micro | Access | Access (introduced) | Accessibility and availability of healthcare services and medication as an outcome of the usage of the HIS. Patient-provider participation or engagement, self-management, access to personal health data, shared decision making, and patient-provider communication as an outcome of the usage of the HIS. | Accessing information (as an activity) in the HIS by patients --> 07-12. Use or 01. Functionalities if it concerns a specific feature. Internet, computer or smartphone access --> 27. Info and infrastructure. Availability or accessibility of information in the system when quality of information of the HIS --> 05. Availability. Service when quality of the HIS --> 06. Service. Patient participation in or engagement with healthcare, or patient participation/engagement not further specified 14. Appropriateness and effectiveness. |
| 20 | Meso | People | Individuals and groups (extended) | ‘Types of individuals/groups that can affect the adoption of an HIS, including patients/clients and families, healthcare providers and managers, policy planners and stakeholder groups’[9]. For each result found we classify the type of individual it concerns in the "subgroup" column. | Characteristics of the individual itself about which the factor is reported --> 21. Personal characteristics. |
| 21 | Meso | People | Personal characteristics (extended) | ‘Degree to which an individual’s characteristics, such as age, gender, education, [socio-economic status, ethnicity, computer skills, (health) literacy, health status,] experience and expertise can affect the adoption of an HIS’[9]. Behaviour. | Competency related to HIS use -> 10 Competency. |
| 22 | Meso | People | Personal expectations (extended) | ‘Degree to which an individual [who can affect the adoption of an HIS] believes HISs are important, can improve job performance, [daily life, health status or quality of life] and that infrastructures exist to support its adoption’[9], or expectation about whether they themselves or others will be able or willing to use the system. Also norms and preferences related to HIS use. | User satisfaction about a particular HIS being used --> 11. Usefulness. Expectations about the adoption of HISs of the public in general or people that cannot affect the adoption of an HIS --> 41-43. Societal, political and economic trends. Security of the particular HIS --> 03. Security. 201705: Use intention to use the particular HIS --> 09. Intention to use |
| 23 | Meso | People | Roles and responsibilities (extended) | ‘Position, function and obligation of an individual/group in relation to HIS adoption, e.g., being a stakeholder, leader, champion and project sponsor’[9]. | Type of individual (e.g. caregiver, provider) --> 20. Individuals and group, fill out in the subgroup column. Characteristics of these individuals --> 21. Personal characteristics. |
| 24 | Meso | Organization | Strategy (unchanged) | ‘Set of coordinated activities designed to achieve the overall mandate and objectives of the organization, including HIS adoption’[9]. |  |
| 25 | Meso | Organization | Culture (extended) | ‘Ingrained set of shared [norms,] values, beliefs and assumptions acquired by members of an organization over time, including their views toward HISs’[9]. |  |
| 26 | Meso | Organization | Structure and processes (unchanged) | ‘Organizational functioning, including governance, configuration, reporting relationships and communication, as well as business and patient care processes such as continuity of care’[9]. |  |
| 27 | Meso | Organization | Info- and infrastructure (extended) | ‘HIS governance/management, technical architectures, information assets, level of integration and privacy/security in place or planned’[9]. Infrastructure in the environment of the consumer (patient or caregiver), including smartphone, internet, computer access. | Belief of an individual that infrastructures exist to support the adoption an HIS --> 22. Personal expectations. Usage of infrastructure --> 21. personal characteristic. |
| 28 | Meso | Organization | Return on value (extended) | ‘Economic return on HIS investment and use in terms of cost benefit, effectiveness, utility and avoidance, business case, return on investment, value propositions and benefits realization’[9]. | Outcomes of usage of the particular HIS --> 13-19. Net benefits. Costs and benefits as an incentive/ determinant on macro level outside of the organisation --> 39. Added values. |
| 29 | Meso | Implementation | Stage (unchanged) | ‘HIS adoption stages – initiation, building/buying, introduction and adaptation’[9]. |  |
| 30 | Meso | Implementation | Project (extended) | ‘Planning, activities and resources for HIS adoption, including scope, objectives, constraints, targets, governance, methodology, commitment, communication, training, risks, monitoring, reporting and expectations’[9]. In the maintenance phase also uptake by new patients can be understood as part of the ongoing project implementation. |  |
| 31 | Meso | Implementation | HIS-practice fit (extended) | ‘Degree of fit between the HIS and organizational work practices [of the care provider or the daily life of the patient or caregiver], and the extent of change from HIS adoption’[9]. |  |
| 32 | Macro | Healthcare standards | HIS standards (unchanged) | ‘Types of data, messaging, terminology and technology standards that influence the healthcare industry as a whole with respect to HIS adoption’[9]. |  |
| 33 | Macro | Healthcare standards | Performance standards (unchanged) | ‘Types of organizational performance standards in place, such as those for accreditation of healthcare facilities and performance targets’[9]. |  |
| 34 | Macro | Healthcare standards | Practice standards (unchanged) | ‘Desired level of professional competency, knowledge, skills and performance in the workplace, including HIS adoption’[9]. |  |
| 35 | Macro | Legislation, policy and governance | Legislative acts (extended) | ‘Types of HIS-related legislative acts, such as health information and privacy laws that govern the adoption of HISs’[9]. | Privacy concerns --> 22. Personal expectation. |
| 36 | Macro | Legislation, policy and governance | Regulations and policies (extended) | ‘Types of HIS-related regulations/policies, such as data access and security/privacy guidelines’[9]. | Privacy concerns --> 22. Personal expectation; Organisational security policy --> 27. Info- and infrastructure; System security --> 03. Security. Organizational policy regarding data access --> 26. Structure and process. |
| 37 | Macro | Legislation, policy and governance | Governance bodies (unchanged) | ‘Types of accountability and decision-making structures in place regarding the adoption of HISs’[9]. |  |
| 38 | Macro | Funding and incentives | Remunerations (unchanged) | ‘Types of compensation available, such as alternative payment schemes to entice change at the individual, practice and organizational levels’[9]. |  |
| 39 | Macro | Funding and incentives | Added values (extended) | ‘General expectations available, such as alternative payment schemes to entice change at the individual, practice and organizational levels’[9]. | Outcomes of usage of the particular HIS --> 13-19. Net benefits. Cost/benefit as an incentive/ determinant on organizational level that influences the HIS adoption --> 28. Return on value. |
| 40 | Macro | Funding and incentives | Incentive programs (unchanged) | ‘Types of reward programs available that entice change at the individual, practice and organizational levels’[9]. |  |
| 41 | Macro | Societal, political and economic trends | Societal trends (extended) | Societal trends or ‘General expectations of the public toward healthcare and HISs’[9]. | Expectations of particular people or groups who have a direct influence on HIS adoption --> 22. Personal expectations. |
| 42 | Macro | Societal, political and economic trends | Political trends (extended) | Political trends or ‘General political climates toward healthcare and HISs’[9]. |  |
| 43 | Macro | Societal, political and economic trends | Economic trends (extended) | Economic trends or ‘General economic investment climates toward healthcare and HISs’[9]. |  |
| 44 | Other | Other | Other (introduced) | Factor does not fit in CAF, other category proposed for this determinant or outcome of the adoption and use of the HIS. |  |

## Table B: Metrics per CAF category

The purpose of this table is to provide an overview of metrics found in the literature that belong to each category. This can be used to classify results in literature reviews. It can also be used in implementation studies to help identifying factors and metrics to evaluate. Reporting results from evaluation studies in the CAF will enable comparison among different studies. This table contains the metrics we found from a thematic analysis of the quotes in the reviews and that we classified into each category. Further details on the procedure to apply the adapted CAF to literature studies can be found in the review protocol that was registered at PROSPERO under CRD42018084542.

Table B Metrics per category with the category Id, level, dimension.

| Id | Level | Dimension | Category | Metrics |
| --- | --- | --- | --- | --- |
| 00 | Exclude | Exclude | Exclude |  |
| 01 | Micro | System quality | Functionality | Access to medical records, Add comments to notes, Adding text to pictures, Alerts, notifications and reminders, Appointment reminders, Appointment scheduling, Assistive technology, Automatic data input, Control over health information, Create own care plan, Data entry, Data exchange, Decision support, Design, Diagnostic tool, Discharge information, E-prescribing, Feature to contact multiple providers, Financial administration, Graphs, Health care services use, Health status management, Help remember daily tasks, Home monitoring, Hospital information, Immunization records, In-patient schedule, Information about daily hospital routine, Information about healthcare team, Information conveyance, Input methods, Integration of PHR with healthcare system, Journal, Lifestyle advice, Medical history, Medical renewal reminders, Medication information, Medication management, Medication renewal, Medication warnings, Medium (e.g. paper, electronic), Missing functionality, More providers in the system, Multimedia, Ordering meals, Patient education, Patient summary, Personalization, Prevention recommendations, Printing, Privacy and security settings, Prognostic tool, Provider emergency access, Proxy access, Record management, Risk assessment tool, Safety information, Secure messaging, Sharing medical information, Symptom checker, Take notes, Technology platform options, Terminology tool, Test results, Tools for health management, Tools to understand health information, Triage of messages, Video calling, Video with use instruction, View billing information, View health information, View lab results, View medication list, View notes, View operative reports, View test results, Voice recording |
| 02 | Micro | System quality | Performance | System response time, Technical issues |
| 03 | Micro | System quality | Security | Audit trail, Authentication, Authorization, Security |
| 04 | Micro | Information quality | Content | Accuracy, Amount of documentation, Coded sensitive information, Consistency, Content, Intelligibility, Language, Not overwhelming, Relatedness of information, Sensitivity, View notes |
| 05 | Micro | Information quality | Availability | Availability, Timeliness |
| 06 | Micro | Service quality | Service | Clinical support, Service response time, Support, Technical support |
| 07 | Micro | Use | Use behaviour/pattern | Adoption, Registration, Use pattern |
| 08 | Micro | Use | Self-reported use |  |
| 09 | Micro | Use | Intention to use | Intention to use |
| 10 | Micro | User satisfaction | Competency | Ability to use the system, Learnability, Skills, System feature awareness |
| 11 | Micro | User satisfaction | Usefulness | Usefulness, User satisfaction |
| 12 | Micro | User satisfaction | Ease of use | Ease of use, Navigation, Task completion |
| 13 | Micro | Care Quality | Patient safety | Error identification, Medical error, Medication error, Patient safety |
| 14 | Micro | Care Quality | Appropriateness and effectiveness | Appointment attendance, Being prepared for emergencies, Effective consultations, Emergency department visits, Health care services use, Immunisation, Inconvenience, Knowledge and understanding about personal health, Manage administrative concerns, Medication adherence, Medication adjustment, Medication management, Number of appointments, Patient participation in health care, Preventive services use, Quality of care, Satisfaction with care, Treatment adherence |
| 15 | Micro | Care Quality | Health outcomes | Confidence, Disease control, Medical outcome, Physiological measures, Psychological outcomes |
| 16 | Micro | Productivity | Efficiency | Appointment length, Customer retention, Efficiency, Number of appointments, Number of hospitalisations, Number of messages, Number of phone contacts, Registration time, Repetition of information, Time savings, Workload (outcome) |
| 17 | Micro | Productivity | Care coordination | Access to information by providers, Care coordination, Continuity of care, Responsibility for record management, Workflow (outcome) |
| 18 | Micro | Productivity | Net cost | Financial benefits |
| 19 | Micro | Access | Access | Access to personal health information, Communication between patients and clinicians, Decision making, Focus on patients, Patient-provider participation, Patient-provider relationship, Preparation for clinical visits, Responsibility for record management, Self-management, Sharing personal health information |
| 20 | Meso | People | Individuals and groups |  |
| 21 | Meso | People | Personal characteristics | Activeness in decision making, Address, Admission status, Adoption, Age, Age of caretaker, Autonomy, Awareness, Behaviour, Being sufficiently informed, Cognition, Computer and internet skills, Computer skills, Computer use, Conceptual knowledge, Confidence, Coping style, Education, EHR use, Employment, Engagement with PHRs, Ethnicity, Feeling responsible for their own health, Feeling sufficiently informed, Gender, Health care use, Health literacy, Health status, Insurance, Internet access, Internet use, Keeping their own medical records, Knowledge, Knowledge about right to access medical records, Language, Lifestyle, Literacy, Marital status, Medication use, Memory, Motor skills, Numeracy, Patient activation, Provider use of PHR, Receiving information from other sources, Receptiveness to information, Sensory and perceptual abilities, Skills, Socioeconomic status, Specialty, Technology use, Time constraints, Trust in healthcare provider, Use of alternative technology |
| 22 | Meso | People | Personal expectations | Adoption by providers, Alternative technologies, Anxiety to learn, Awareness of PHR, Beliefs, Choice for a provider, Competitiveness, Concerns, Expectations, Fear for anguish through consulting records, Fear of not understanding medical records, Intention to use, Interest in PHRs, Need to keep their own medical records, Needs, Norms, Patient safety, Perceived PHR value, Preferences, Privacy concerns, Skepticism, Willingness to pay |
| 23 | Meso | People | Roles and responsibilities |  |
| 24 | Meso | Organization | Strategy |  |
| 25 | Meso | Organization | Culture | Norms |
| 26 | Meso | Organization | Structure and processes | Care process, Cooperation with healthcare provider, Involvement of healthcare team, Letting patients exchange medical data, Patient-provider relationship, Practice size, Process, Setting, Social environment, Staffing, Time constraints, Workload (determinant) |
| 27 | Meso | Organization | Info- and infrastructure | Authentication mechanisms, Authorization policy, Computer access, Data exchange infrastructure, Data governance, Hardware, Infrastructure, Integration of PHR with healthcare system, Internet access, Internet and computer access, Internet speed, Interoperability, Kiosks, Security measures, Smartphone access |
| 28 | Meso | Organization | Return on value | PHR value |
| 29 | Meso | Implementation | Stage |  |
| 30 | Meso | Implementation | Project | Communication about PHR, Demonstration, Design decisions, Family recommendation, Participatory design, Provider endorsement, Recommendations, Registration reminders, Training, Trial period, Usability testing |
| 31 | Meso | Implementation | HIS-practice fit | HIS-practice fit |
| 32 | Macro | Healthcare standards | HIS standards | HIS standards, Interoperability |
| 33 | Macro | Healthcare standards | Performance standards |  |
| 34 | Macro | Healthcare standards | Practice standards |  |
| 35 | Macro | Legislation, policy and governance | Legislative acts |  |
| 36 | Macro | Legislation, policy and governance | Regulations and policies | Regulation |
| 37 | Macro | Legislation, policy and governance | Governance bodies |  |
| 38 | Macro | Funding and incentives | Remunerations | Reimbursement |
| 39 | Macro | Funding and incentives | Added values | Added value |
| 40 | Macro | Funding and incentives | Incentive programs | Incentive programs |
| 41 | Macro | Societal, political and economic trends | Societal trends | Education curricula, Societal trends |
| 42 | Macro | Societal, political and economic trends | Political trends |  |
| 43 | Macro | Societal, political and economic trends | Economic trends |  |
| 44 | Other | Other | Other | Regional health information exchange infrastructure |

## Table C: Agreement per CAF category

Table C Number of times a category was chosen by a reviewer: with category, the total number of times n it was chosen by a reviewer, and the number of times n and percentage % agreement and disagreement

| Category | n total | n (%) agree |
| --- | --- | --- |
| 00. Exclude | 22 | 0 (0) |
| 01. Functionality | 376 | 194 (51.6) |
| 02. Performance | 0 |  |
| 03. Security | 1 | 0 (0) |
| 04. Content | 40 | 10 (25) |
| 05. Availability | 7 | 4 (57.1) |
| 06. Service | 17 | 8 (47.1) |
| 07. Use behaviour/pattern | 660 | 474 (71.8) |
| 08. Self-reported use | 3 | 0 (0) |
| 09. Intention to use | 10 | 2 (20) |
| 10. Competency | 50 | 22 (44) |
| 11. Usefulness | 73 | 52 (71.2) |
| 12. Ease of use | 38 | 22 (57.9) |
| 13. Patient safety | 20 | 16 (80) |
| 14. Appropriateness and effectiveness | 248 | 158 (63.7) |
| 15. Health outcomes | 34 | 10 (29.4) |
| 16. Efficiency | 81 | 70 (86.4) |
| 17. Care coordination | 9 | 4 (44.4) |
| 18. Net cost | 1 | 0 (0) |
| 19. Access | 109 | 58 (53.2) |
| 20. Individuals and groups | 0 |  |
| 21. Personal characteristics | 412 | 350 (85) |
| 22. Personal expectations | 208 | 144 (69.2) |
| 23. Roles and responsibilities | 0 |  |
| 24. Strategy | 0 |  |
| 25. Culture | 0 |  |
| 26. Structure and processes | 7 | 0 (0) |
| 27. Info- and infrastructure | 17 | 6 (35.3) |
| 28. Return on value | 1 | 0 (0) |
| 29. Stage | 0 |  |
| 30. Project | 48 | 12 (25) |
| 31. HIS-practice fit | 1 | 0 (0) |
| 32. HIS standards | 0 |  |
| 33. Performance standards | 0 |  |
| 34. Practice standards | 0 |  |
| 35. Legislative acts | 0 |  |
| 36. Regulations and policies | 0 |  |
| 37. Governance bodies | 0 |  |
| 38. Remunerations | 0 |  |
| 39. Added values | 0 |  |
| 40. Incentive programs | 2 | 0 (0) |
| 41. Societal trends | 0 |  |
| 42. Political trends | 0 |  |
| 43. Economic trends | 0 |  |
| 44. Other | 1 | 0 (0) |

## Table D: Agreement per CAF category sorted by percentage agreement

Table D Number of times a category was chosen by a reviewer: with category, the total number of times n it was chosen by a reviewer, and the number of times n and percentage % agreement and disagreement. Sorted by descending percentage agreement.

| Category | n total | n (%) agree |
| --- | --- | --- |
| 16. Efficiency | 81 | 70 (86.4) |
| 21. Personal characteristics | 412 | 350 (85) |
| 13. Patient safety | 20 | 16 (80) |
| 07. Use behaviour/pattern | 660 | 474 (71.8) |
| 11. Usefulness | 73 | 52 (71.2) |
| 22. Personal expectations | 208 | 144 (69.2) |
| 14. Appropriateness and effectiveness | 248 | 158 (63.7) |
| 12. Ease of use | 38 | 22 (57.9) |
| 05. Availability | 7 | 4 (57.1) |
| 19. Access | 109 | 58 (53.2) |
| 01. Functionality | 376 | 194 (51.6) |
| 06. Service | 17 | 8 (47.1) |
| 17. Care coordination | 9 | 4 (44.4) |
| 10. Competency | 50 | 22 (44) |
| 27. Info- and infrastructure | 17 | 6 (35.3) |
| 15. Health outcomes | 34 | 10 (29.4) |
| 04. Content | 40 | 10 (25) |
| 30. Project | 48 | 12 (25) |
| 09. Intention to use | 10 | 2 (20) |
| 00. Exclude | 22 | 0 (0) |
| 03. Security | 1 | 0 (0) |
| 08. Self-reported use | 3 | 0 (0) |
| 18. Net cost | 1 | 0 (0) |
| 26. Structure and processes | 7 | 0 (0) |
| 28. Return on value | 1 | 0 (0) |
| 31. HIS-practice fit | 1 | 0 (0) |
| 40. Incentive programs | 2 | 0 (0) |
| 44. Other | 1 | 0 (0) |
| 02. Performance | 0 |  |
| 20. Individuals and groups | 0 |  |
| 23. Roles and responsibilities | 0 |  |
| 24. Strategy | 0 |  |
| 25. Culture | 0 |  |
| 29. Stage | 0 |  |
| 32. HIS standards | 0 |  |
| 33. Performance standards | 0 |  |
| 34. Practice standards | 0 |  |
| 35. Legislative acts | 0 |  |
| 36. Regulations and policies | 0 |  |
| 37. Governance bodies | 0 |  |
| 38. Remunerations | 0 |  |
| 39. Added values | 0 |  |
| 41. Societal trends | 0 |  |
| 42. Political trends | 0 |  |
| 43. Economic trends | 0 |  |

1. Abbreviations: CAF, Clinical Adoption Framework; EHR, Electronic Health Record; HIS, Health Information System; PHR, Personal Health Record

   Corresponding author at: Amsterdam UMC, University of Amsterdam, Department of Medical Informatics, Amsterdam Public Health, Room J1b-109, Meibergdreef 9, P.O. Box 22700, 1100 DE, Amsterdam, The Netherlands. E-mail address: [h.j.vanmens@amsterdamumc.nl](mailto:h.j.vanmens@amsterdamumc.nl) (Hugo van Mens). [↑](#footnote-ref-1)
2. DOI: 10.12927/hcq.2011.22157, URL: http://www.longwoods.com/content/22157 [↑](#footnote-ref-2)
